# 3-Dimethyl­amino-1-(4-methyl­phen­yl)prop-2-en-1-one

**DOI:** 10.1107/S1600536809048879

**Published:** 2009-12-04

**Authors:** Juean Deng, Dongsheng Shen, Zongzhou Zhou

**Affiliations:** a College of Chemistry and Chemical Engineering, Guangdong Pharmaceutical University, Guangzhou 510006, People’s Republic of China

## Abstract

In the title compound, C_12_H_15_NO, the C=C and C=O functional groups and the benzene ring are involved in an extended conjugated system. The mol­ecules are essentially planar with a maximal deviation from planarity for the non-H atoms of 0.062 (2) Å.

## Related literature

For the pharmaceutical activity of enamino­nes, see: Edafiogh *et al.* (2003 ▶); Eddington *et al.* (2003 ▶). For the use of enamino­nes as chelating ligands for main group metals and transition metals in coordination chemistry, see: Cindrić *et al.* (2004 ▶); Shi *et al.* (2008 ▶). For the chemical synthesis of enamino­nes, see: Kantevari *et al.* (2007 ▶); Ke *et al.* (2009 ▶). For the crystal structures of enamino­nes, see: Lemmerer *et al.* (2007 ▶); Bertolasi *et al.* (1999 ▶); Blake *et al.* (1996 ▶).
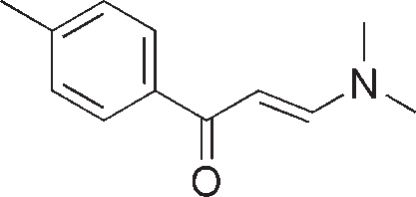


## Experimental

### 

#### Crystal data

C_12_H_15_NO
*M* *_r_* = 189.25
Monoclinic, 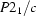

*a* = 8.7918 (17) Å
*b* = 5.9506 (12) Å
*c* = 20.789 (4) Å
β = 99.300 (3)°
*V* = 1073.3 (4) Å^3^
*Z* = 4
Mo *K*α radiation
μ = 0.07 mm^−1^
*T* = 173 K
0.08 × 0.06 × 0.03 mm

#### Data collection

Bruker APEXII CCD diffractometer
Absorption correction: multi-scan (*SADABS*; Sheldrick, 2004 ▶) *T*_min_ = 0.994, *T*_max_ = 0.998
5180 measured reflections
2290 independent reflections
1731 reflections with *I* > 2σ(*I*)
*R*_int_ = 0.023

#### Refinement

*R*[*F* ^2^ > 2σ(*F* ^2^)] = 0.046
*wR*(*F* ^2^) = 0.150
*S* = 1.02
2290 reflections
130 parameters
H-atom parameters constrained
Δρ_max_ = 0.27 e Å^−3^
Δρ_min_ = −0.23 e Å^−3^

### 

Data collection: *APEX2* (Bruker, 2005 ▶); cell refinement: *SAINT-Plus* (Bruker, 2005 ▶); data reduction: *SAINT-Plus*; program(s) used to solve structure: *SHELXS97* (Sheldrick, 2008 ▶); program(s) used to refine structure: *SHELXL97* (Sheldrick, 2008 ▶); molecular graphics: *SHELXTL* (Sheldrick, 2008 ▶); software used to prepare material for publication: *SHELXTL*.

## Supplementary Material

Crystal structure: contains datablocks I, global. DOI: 10.1107/S1600536809048879/vm2013sup1.cif

Structure factors: contains datablocks I. DOI: 10.1107/S1600536809048879/vm2013Isup2.hkl

Additional supplementary materials:  crystallographic information; 3D view; checkCIF report

## Figures and Tables

**Fig. 1.**
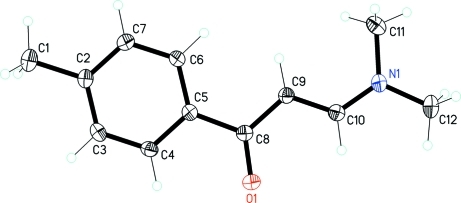
The structure of the title compound, with displacement ellipsoids at the 30% probability level.

## supplementary crystallographic information

### Comment

Enaminones and their metal complexes have been widely studied due to their applications in the fields of optical chemistry, medicinal chemistry and biotechnology. Those ligands are versatile synthetic intermediates that combine the ambident nucleophilicity of enamines with the ambident electrophilicity of enones and have been extensively used for the preparation of a variety of heterocyclic systems including some natural products and analogues. Moreover, in coordination chemistry, enaminones can be used as good chelating ligands for main group metals and transition metals (Cindrić *et al.*, 2004; Shi *et al.*,2008). We report here the synthesis and structure of the title compound. The molecular structure of the title compound is shown in Fig.1. The molecule crystallized as an E isomer with extended conjugation involving N, C=C, C=O, and the benzene ring. As a consequence the molecule is planar, the maximal deviation from planarity for the non-hydrogen atoms is 0.062 (2) Å.

### Experimental

A solution of 4-Methylacetophenone (13.2 g, 0.1 mol) in ethyl formate (14.8 g, 0.2 mol) was added dropwise to a stirred suspension of sodium ethoxide(6.8 g, 0.1 mol) in anhydrous diethyl ether (50 ml) at room temperature. After stirring for 4 h, 8.8 g Dimethylamine hydrochloride (0.11 mol) in 20 ml water was added dropwise to the stirred suspended matter, and it was stirred for another 2 h. Then, the organic phase was separated, and the solvent was removed on a rotary evaporator, the residual was recrystallized in hexane-acetone (10:1) in an afford of the title compound (15.2 g). Crystals were obtained by slow evaporation of a solution in diethyl ether at room temperature.

### Refinement

All H atoms were placed in geometrically idealized positions and constrained to ride on their parent atoms with C—H distances in the range 0.95–0.98 Å.

### Crystal data

**Table:**

C_12_H_15_NO	*F*(000) = 408
*M**_r_* = 189.25	*D*_x_ = 1.171 Mg m^−^^3^
Monoclinic, *P*2_1_/*c*	Melting point: 367 K
Hall symbol: -P 2ybc	Mo *K*α radiation, λ = 0.71073 Å
*a* = 8.7918 (17) Å	Cell parameters from 1291 reflections
*b* = 5.9506 (12) Å	θ = 3.1–28.6°
*c* = 20.789 (4) Å	µ = 0.07 mm^−^^1^
β = 99.300 (3)°	*T* = 173 K
*V* = 1073.3 (4) Å^3^	Plate, colourless
*Z* = 4	0.08 × 0.06 × 0.03 mm

### Data collection

**Table:**

Bruker APEX CCD diffractometer	2290 independent reflections
Radiation source: fine-focus sealed tube	1731 reflections with *I* > 2σ(*I*)
graphite	*R*_int_ = 0.023
ω and phi scans	θ_max_ = 27.0°, θ_min_ = 2.0°
Absorption correction: multi-scan (*SADABS*; Sheldrick, 2004)	*h* = −10→10
*T*_min_ = 0.994, *T*_max_ = 0.998	*k* = −7→7
5180 measured reflections	*l* = −11→26

### Refinement

**Table:**

Refinement on *F*^2^	Primary atom site location: structure-invariant direct methods
Least-squares matrix: full	Secondary atom site location: difference Fourier map
*R*[*F*^2^ > 2σ(*F*^2^)] = 0.046	Hydrogen site location: inferred from neighbouring sites
*wR*(*F*^2^) = 0.150	H-atom parameters constrained
*S* = 1.02	*w* = 1/[σ^2^(*F*_o_^2^) + (0.0918*P*)^2^ + 0.1894*P*] where *P* = (*F*_o_^2^ + 2*F*_c_^2^)/3
2290 reflections	(Δ/σ)_max_ < 0.001
130 parameters	Δρ_max_ = 0.27 e Å^−^^3^
0 restraints	Δρ_min_ = −0.23 e Å^−^^3^

### Special details

**Table:**

Geometry. All e.s.d.'s (except the e.s.d. in the dihedral angle between two l.s. planes) are estimated using the full covariance matrix. The cell e.s.d.'s are taken into account individually in the estimation of e.s.d.'s in distances, angles and torsion angles; correlations between e.s.d.'s in cell parameters are only used when they are defined by crystal symmetry. An approximate (isotropic) treatment of cell e.s.d.'s is used for estimating e.s.d.'s involving l.s. planes.
Refinement. Refinement of *F*^2^ against ALL reflections. The weighted *R*-factor *wR* and goodness of fit *S* are based on *F*^2^, conventional *R*-factors *R* are based on *F*, with *F* set to zero for negative *F*^2^. The threshold expression of *F*^2^ > σ(*F*^2^) is used only for calculating *R*-factors(gt) *etc*. and is not relevant to the choice of reflections for refinement. *R*-factors based on *F*^2^ are statistically about twice as large as those based on *F*, and *R*- factors based on ALL data will be even larger.

### Fractional atomic coordinates and isotropic or equivalent isotropic displacement parameters (Å^2^)

**Table:**

	*x*	*y*	*z*	*U*_iso_*/*U*_eq_	
O1	0.19626 (13)	0.51500 (19)	0.07917 (6)	0.0490 (4)	
N1	−0.15817 (14)	0.0530 (2)	0.05528 (6)	0.0327 (3)	
C1	0.84154 (18)	0.1518 (3)	0.24118 (8)	0.0439 (4)	
H1A	0.8475	−0.0002	0.2596	0.066*	
H1B	0.8626	0.2619	0.2765	0.066*	
H1C	0.9179	0.1680	0.2120	0.066*	
C2	0.68222 (16)	0.1912 (2)	0.20336 (7)	0.0308 (3)	
C3	0.64246 (17)	0.3950 (3)	0.17274 (7)	0.0346 (4)	
H3A	0.7179	0.5101	0.1744	0.042*	
C4	0.49477 (17)	0.4341 (2)	0.13971 (7)	0.0318 (3)	
H4A	0.4705	0.5753	0.1193	0.038*	
C5	0.38156 (16)	0.2684 (2)	0.13621 (6)	0.0271 (3)	
C6	0.42232 (16)	0.0632 (2)	0.16573 (7)	0.0302 (3)	
H6A	0.3478	−0.0535	0.1632	0.036*	
C7	0.57032 (17)	0.0256 (3)	0.19896 (7)	0.0328 (4)	
H7A	0.5952	−0.1161	0.2190	0.039*	
C8	0.22148 (16)	0.3234 (2)	0.10167 (7)	0.0301 (3)	
C9	0.10551 (16)	0.1520 (2)	0.09670 (7)	0.0291 (3)	
H9A	0.1299	0.0087	0.1157	0.035*	
C10	−0.03947 (16)	0.1940 (2)	0.06473 (7)	0.0296 (3)	
H10A	−0.0578	0.3408	0.0472	0.036*	
C11	−0.1441 (2)	−0.1751 (3)	0.07988 (9)	0.0442 (4)	
H11A	−0.0440	−0.2368	0.0739	0.066*	
H11B	−0.2268	−0.2675	0.0560	0.066*	
H11C	−0.1520	−0.1752	0.1264	0.066*	
C12	−0.30880 (17)	0.1211 (3)	0.02142 (8)	0.0411 (4)	
H12A	−0.3026	0.2740	0.0045	0.062*	
H12B	−0.3833	0.1175	0.0518	0.062*	
H12C	−0.3421	0.0178	−0.0148	0.062*	

### Atomic displacement parameters (Å^2^)

**Table:**

	*U*^11^	*U*^22^	*U*^33^	*U*^12^	*U*^13^	*U*^23^
O1	0.0387 (7)	0.0328 (6)	0.0712 (9)	−0.0016 (5)	−0.0038 (6)	0.0170 (6)
N1	0.0276 (6)	0.0359 (7)	0.0341 (7)	−0.0024 (5)	0.0035 (5)	−0.0010 (5)
C1	0.0330 (9)	0.0542 (10)	0.0421 (9)	0.0026 (7)	−0.0008 (7)	−0.0023 (8)
C2	0.0276 (7)	0.0373 (8)	0.0274 (7)	0.0008 (6)	0.0047 (5)	−0.0040 (6)
C3	0.0325 (8)	0.0346 (8)	0.0367 (8)	−0.0090 (6)	0.0056 (6)	−0.0041 (6)
C4	0.0360 (8)	0.0261 (7)	0.0335 (8)	−0.0030 (6)	0.0065 (6)	0.0026 (6)
C5	0.0283 (7)	0.0286 (7)	0.0250 (7)	−0.0013 (5)	0.0062 (5)	−0.0018 (5)
C6	0.0299 (7)	0.0280 (7)	0.0326 (8)	−0.0030 (6)	0.0050 (6)	0.0013 (6)
C7	0.0356 (8)	0.0318 (8)	0.0309 (7)	0.0028 (6)	0.0048 (6)	0.0031 (6)
C8	0.0321 (8)	0.0280 (7)	0.0305 (7)	0.0006 (6)	0.0056 (6)	0.0015 (6)
C9	0.0286 (8)	0.0282 (7)	0.0307 (7)	0.0004 (6)	0.0055 (6)	0.0019 (6)
C10	0.0316 (8)	0.0288 (7)	0.0296 (7)	−0.0003 (6)	0.0083 (6)	−0.0007 (6)
C11	0.0457 (10)	0.0360 (9)	0.0512 (10)	−0.0103 (7)	0.0090 (8)	−0.0011 (7)
C12	0.0285 (8)	0.0562 (11)	0.0377 (8)	−0.0011 (7)	0.0031 (6)	−0.0056 (7)

### Geometric parameters (Å, °)

**Table:**

O1—C8	1.2388 (18)	C5—C8	1.509 (2)
N1—C10	1.3288 (18)	C6—C7	1.389 (2)
N1—C11	1.448 (2)	C6—H6A	0.9500
N1—C12	1.453 (2)	C7—H7A	0.9500
C1—C2	1.510 (2)	C8—C9	1.434 (2)
C1—H1A	0.9800	C9—C10	1.362 (2)
C1—H1B	0.9800	C9—H9A	0.9500
C1—H1C	0.9800	C10—H10A	0.9500
C2—C7	1.385 (2)	C11—H11A	0.9800
C2—C3	1.388 (2)	C11—H11B	0.9800
C3—C4	1.387 (2)	C11—H11C	0.9800
C3—H3A	0.9500	C12—H12A	0.9800
C4—C5	1.395 (2)	C12—H12B	0.9800
C4—H4A	0.9500	C12—H12C	0.9800
C5—C6	1.387 (2)		
			
C10—N1—C11	121.29 (13)	C2—C7—C6	121.09 (13)
C10—N1—C12	121.85 (13)	C2—C7—H7A	119.5
C11—N1—C12	116.84 (13)	C6—C7—H7A	119.5
C2—C1—H1A	109.5	O1—C8—C9	123.07 (13)
C2—C1—H1B	109.5	O1—C8—C5	118.41 (13)
H1A—C1—H1B	109.5	C9—C8—C5	118.52 (12)
C2—C1—H1C	109.5	C10—C9—C8	120.19 (13)
H1A—C1—H1C	109.5	C10—C9—H9A	119.9
H1B—C1—H1C	109.5	C8—C9—H9A	119.9
C7—C2—C3	117.91 (13)	N1—C10—C9	127.35 (14)
C7—C2—C1	120.88 (14)	N1—C10—H10A	116.3
C3—C2—C1	121.21 (14)	C9—C10—H10A	116.3
C4—C3—C2	121.32 (13)	N1—C11—H11A	109.5
C4—C3—H3A	119.3	N1—C11—H11B	109.5
C2—C3—H3A	119.3	H11A—C11—H11B	109.5
C3—C4—C5	120.69 (14)	N1—C11—H11C	109.5
C3—C4—H4A	119.7	H11A—C11—H11C	109.5
C5—C4—H4A	119.7	H11B—C11—H11C	109.5
C6—C5—C4	117.92 (13)	N1—C12—H12A	109.5
C6—C5—C8	123.77 (13)	N1—C12—H12B	109.5
C4—C5—C8	118.31 (13)	H12A—C12—H12B	109.5
C5—C6—C7	121.06 (13)	N1—C12—H12C	109.5
C5—C6—H6A	119.5	H12A—C12—H12C	109.5
C7—C6—H6A	119.5	H12B—C12—H12C	109.5
